# The effect of mint addition on the physicochemical and organoleptic properties of strawberry sorbets

**DOI:** 10.1016/j.fochx.2025.102271

**Published:** 2025-02-12

**Authors:** Rita Székelyhidi, Erika Lakatos, Zsófia Tóth, Beatrix Sik

**Affiliations:** Department of Food Science, Albert Kázmér Faculty of Agricultural and Food Sciences of Széchenyi István University in Mosonmagyaróvár, Széchenyi István University, Lucsony Street 15-17, 9200 Mosonmagyaróvár, Hungary

**Keywords:** Strawberry sorbet, Mint supplementation, Antioxidant properties, Organic acids, Sugars, Consumer acceptance

## Abstract

The study aimed to produce, analyse, and evaluate the consumer acceptance of a functional peppermint-, and spearmint-enriched (0.5,1,2 %) sorbet, which contains only natural substances in its composition, to meet today's popular health-conscious consumer trends. Regarding acid composition, the sorbets contained malic acid, succinic acid, and citric acid. Sorbets regarded of water-soluble sugars, contained sucrose in the lowest concentration, followed by glucose, and the amount of fructose, which is typical for fruits, was the highest. The sorbets' TPC and TAC contents were determined to be between 510.72 and 743.77 mg GAE/kg, and 906.64 and 1137.67 mg AAE/kg respectively. The average melting rate of the desserts was 0.16–0.22 g/min, and based on consumer acceptance, the control and the samples added with 0.5 % spearmint and peppermint mint were the most favorable. The sorbets containing 1 % and 2 % mint had too intense menthol flavor, thus the enjoyment value of the products was reduced based on consumer feedback.

## Highlights

• Functional strawberry sorbet with mint, and honey supplementation.
• Measurement of functional chemical properties of strawberry-mint sorbet.
• Consumer acceptance of strawberry sorbet prepared with mint addition.

## Graphical abstract

**Figure:**
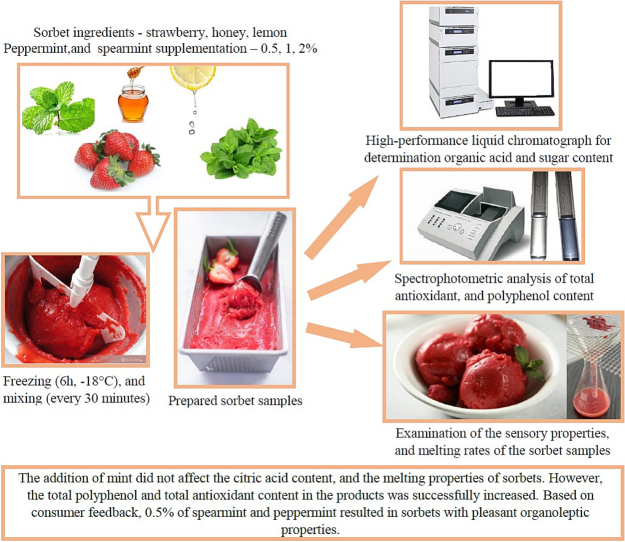

## 1 Introduction

Customer preferences and eating habits are expected to change, resulting in a significant increase in the frozen food market in Europe in the coming years. Between 2022 and 2027, the market is expected to grow by an estimated USD 48.81 billion due to a significant increase in consumer demand (Szydłowska et al., 2024).

One of the best ways to maintain berries' flavor, color, phenolic and antioxidant qualities, and bioactive compounds is freezing (Lilia et al., 2020; Neri et al., 2020; Agnieszka & Wilczyńska, 2023). The conditions of the freezing process significantly influence the quality of frozen products, the method of storage, and the physicochemical properties of the raw materials. Freezing enables the production of new types of fruit products, such as ice creams and sorbet (Góral et al., 2018). Sorbets are frozen desserts that, in the case of industrial production, mainly contain fruit, sugar, water, and stabilizers (Kamińska-Dwórznicka et al., 2019). In contrast to artisanal sorbets, industrially produced sorbets contain more water, added sugar, and stabilizers to reduce costs. Artisan sorbets are typically made using fresh fruit and do not contain stabilizers, so they can only be kept in household freezers for 2–3 days, while they can be stored at temperatures below −18 °C for up to six months (Agnieszka & Wilczyńska, 2023). Its popularity stems from its low calorie content (60–120 kcal/ 100 g), furthermore, they are an appropriate type of ice cream for people who are allergic or intolerant to the ingredients in milk-based products (Agnieszka & Skotnicka, 2022), or follow a vegetarian/ vegan diet (Petkova et al., 2022). The strawberry is an excellent raw material of sorbets because of its anticarcinogenic bioactive compounds, like vitamin C, anthocyanins, and phenolic compounds (Oliveira et al., 2015); regardless of the preservation method (freezing or pasteurization), the fruit can retain significant antioxidant capacity (Agnieszka & Wilczyńska, 2023). The food industry and medicine often use species from the *Lamiaceae* family, such as mint, because of their significant antioxidant qualities. The leaves of the different types of mint have strong antioxidant and free radical-scavenging qualities, primarily because of phenolic acids and flavonoids (Park, 2011). Because of their positive physiological effects, mint leaves can be used as health-protective additives in functional food products (Brown et al., 2019). Due to aromatic compounds and secondary metabolites, fresh or dried leaves of mint are used as flavouring and cooling agents in tea, confectionaries, candies, chewing gums, and different types of frozen desserts (Patil et al., 2018; Saqib et al., 2022). In ice creams, menthol from mint species increases the products' acidity, dry matter, and protein content while decreasing their fat content. The viscosity and overrun of frozen desserts can be decreased by adding mint (Patil et al., 2018).

Natural, additive-free foods are becoming more popular, as are special diets (vegetarian, vegan), and consumers with food allergies and intolerances are calling for non-dairy frozen desserts that are safe to eat and have health benefits (antioxidant, anticarcinogenic, immunomodulatory, anti-inflammatory, antiallergenic, antiviral, and antibacterial etc.) due to their added value. Therefore the aim of the study was to develop a strawberry-based mint-supplemented functional sorbet recipe without water, and stabilizer addition. Furthermore, also the aim was to use analytical methods to prove the beneficial physiological effects of the raw materials and sorbet samples, as well as to assess consumer acceptance of sorbets with organoleptic testing.

## 2 Materials and methods

### 2.1 Chemicals

Malic acid (99.5 %), succinic acid (99.5 %), citric acid (99.5 %), glucose (99.5 %), fructose (99 %), and sucrose (99.5 %) were purchased from Sigma-Aldrich (Budapest, Hungary) to determine the quantity of organic acids and sugars with high-performance liquid chromatography (HPLC) methods.

Chemicals for the determination of polyphenol, and antioxidant content were anhydrous sodium carbonate (99 %, Riedel-de Hӓen, Seelze, Germany), Folin-Ciocalteu reagent (98 %, Sigma-Aldrich, Budapest, Hungary), 2–4-6-tripyridyl-*s*-triazine (TPTZ) (98 %, Sigma-Aldrich, Budapest, Hungary), acetic acid (96 %, Reanal, Budapest, Hungary), anhydrous iron chloride (98 %, Merck, Budapest, Hungary), ascorbic acid (96 %, Sigma-Aldrich, Budapest, Hungary), and gallic acid (98 %, Sigma-Aldrich, Budapest, Hungary).

### 2.2 Sorbet making process

Strawberry and acacia honey were purchased from producers' market (Tata, Hungary), dried peppermint (*Mentha* × *piperita* L.) and spearmint (*Mentha spicata* L.) were obtained from a herbarium (Tata, Hungary), while lemons were provided from a supermarket (Tata, Hungary). To ensure that the addition of mint does not result in an unpleasant sorbet texture, the dried mint samples were chopped with a coffee grinder (Sencor, SCG 2050RDF, Fast Hungary Kft, Szigetszentmiklós, Hungary). For the experiments, the control sorbets were prepared without mint addition, while the experimental sorbets were supplemented with 0.5, 1, and 2 % dried peppermint and spearmint powder.

During the preparation of the sorbets, a syrup was first made from 150 g of honey and 240 mL of water, to which were added 500 g of strawberries, and in the case of the experimental sorbets, 0.5, 1 and 2 % of dried peppermint or spearmint powder. The mixture was crushed with a stick blender (Bosch MSM 2620B, Gerlingen, Germany) until a uniform texture was obtained and the samples were placed in plastic jars in a freezer (Gorenje FN617EEW5, Velenje, Slovenia) at −18 °C. Freezing lasted for 6 h, during the process sorbets were stirred every 30 min with a stick blender to achieve a creamier texture. The same procedure was followed to prepare the control samples, but no herbal supplement was used.

### 2.3 Sample preparation for analytical examinations

The active compounds had to be extracted from the matrix with solvent extraction (Sik et al., 2023) to determine the amounts of organic acids, sugars antioxidants, and polyphenols from strawberry, and mint species. On an analytical balance (Sartorius TE214S, Budapest, Hungary), 20 g of strawberry, and 0.5 g of ground mint samples were weighed into 250 mL Erlenmeyer flasks for extraction. Then, 100 mL of an extraction solution containing methanol, ultra-high purity water (70,30 *V*/V%), and 0,1 mL 37 % hydrochloric acid was added. Elpan 358 S (Katowice, Poland) laboratory shaker at 120 RPM was used for the extraction, which was carried out at room temperature for 1 h. The lemon juice was squeezed and diluted tenfold with ultra-high purity water, and the acacia honey was also diluted in this proportion for the analytical tests. The frozen sorbet samples were thawed, and the samples were used undiluted to test the total polyphenol content (TPC) and organic acid composition, while a fourfold dilution was used to determine the total antioxidant content (TAC) and a tenfold dilution was used to determine the sugar composition. The sorbet samples were diluted with ultra-high purity water. The extracts and dilutions were centrifuged (Hermle Z206 A, Wehingen, Germany) for 20 min at 6000 RPM at room temperature, and the supernatant was further analysed.

### 2.4 HPLC determination conditions of organic acids and sugars

**Fig. 1:**
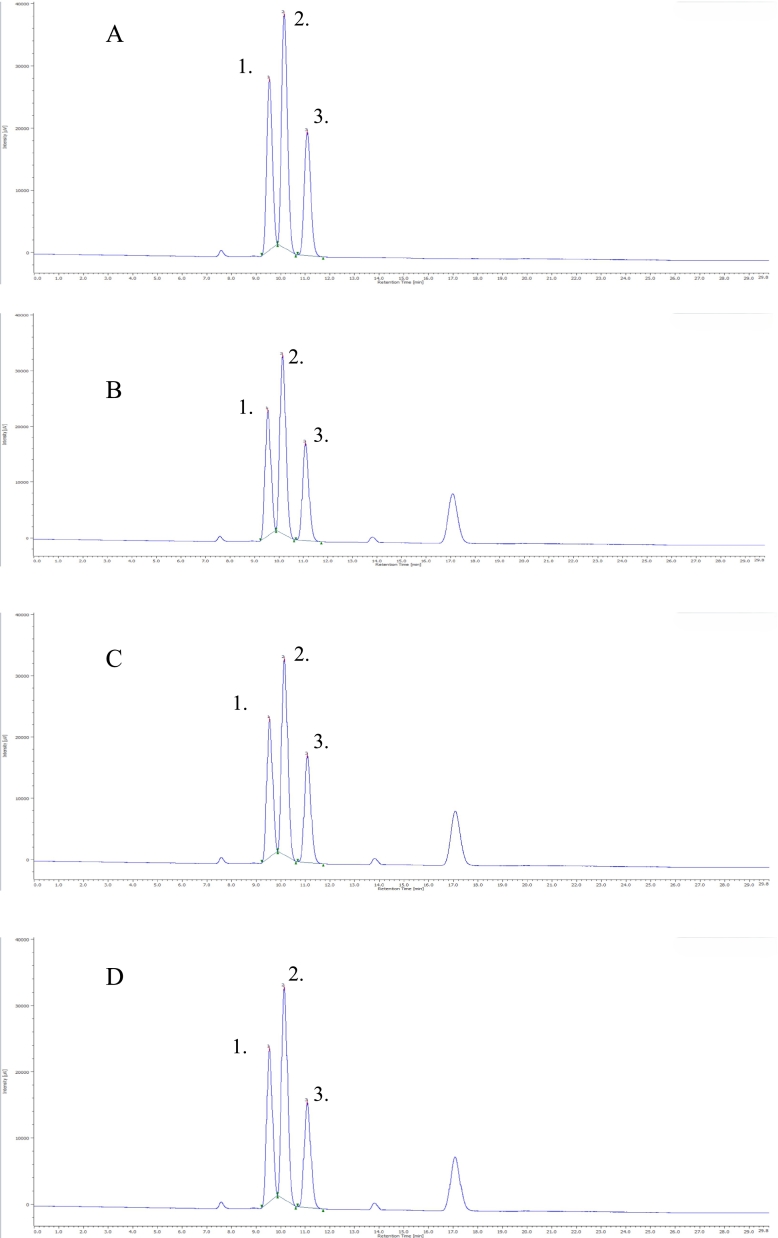
Obtained chromatogram of the 1 mg/mL standard mixture (A), and chromatograms of some sorbet samples (control sorbet - B, peppermint sorbet 0.5 %- C, spearmint sorbet 0.5 % – D) at 210 nm. Citric acid (1), Malic acid (2), Succinic acid (3).

The HPLC analysis procedure was based on the method described by Székelyhidi et al. (2024). For the determination, the supernatant of the samples was filtered into 1.8 mL HPLC vials with a 0.22 μm hydrophilic syringe filter. The Jasco HPLC system used for the analysis of sugar composition consisted of the following units: PU-980 pump, AS-2055 autosampler, DG-1580-53 degassing unit, Merck RI 71 refractive index detector, Supelcogel H (Sigma-Aldrich) column. The column was placed in a 35 °C thermostat (Jones Chromatography Model 7955), and the eluent (ultra-high purity water) flow rate was 0.5 mL/min. The HPLC equipment was calibrated with a series of measuring solutions with a concentration of 0.5–10 mg/mL. The chromatograms are shown in Fig. 1.

**Fig. 2:**
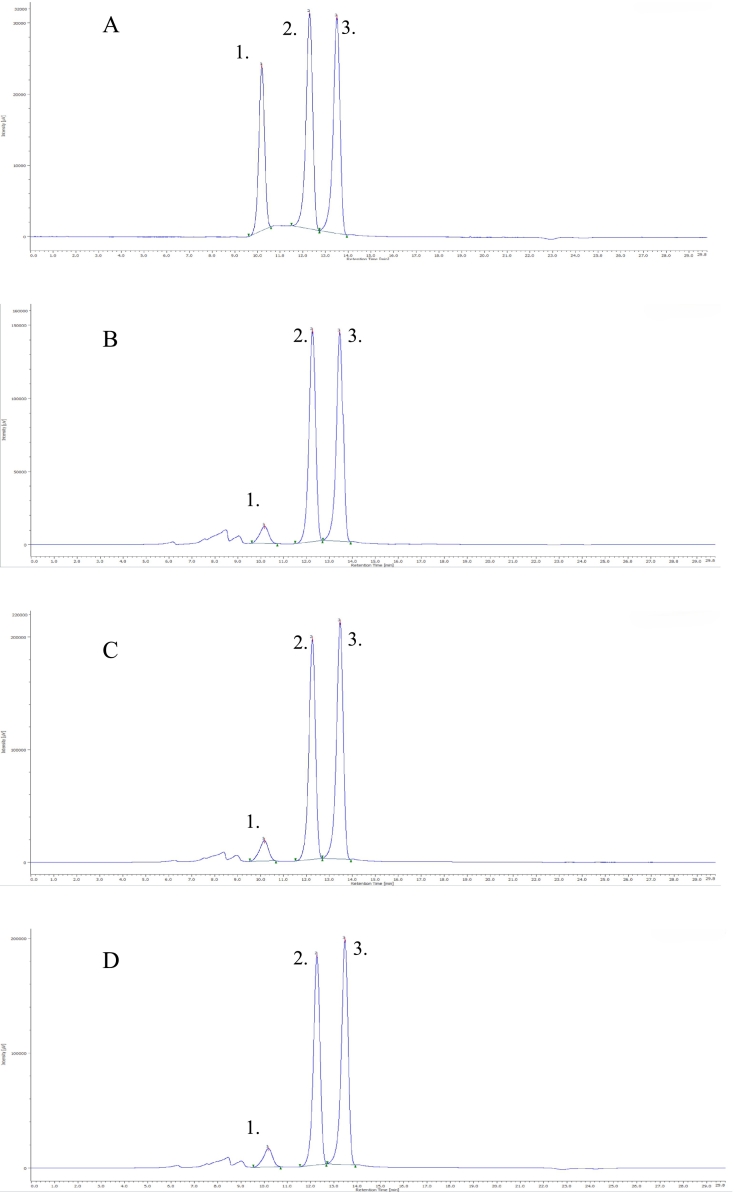
Obtained chromatogram of the 1 mg/mL standard mixture (A), and chromatograms of some sorbet samples (control sorbet - B, peppermint sorbet 0.5 %- C, spearmint sorbet 0.5 % – D). Saccharose (1), Glucose (2), Fructose (3).

The Jasco system was also used for the analysis of organic acids with the following units: PU-980 pump, AS-2055 autosampler, DG-1580-53 degassing unit, UV-975 detector, Jones Chromatography Model 7955 column thermostat, Bio-Rad Aminex HPX-87H column, which also carried out the separation at 35 °C, the eluent was 0.1 % H_2_SO_4_ solution with 0.5 m/min flow rate. The HPLC system was calibrated with a series of measuring solutions with a concentration of 0.05–1 mg/mL. The chromatograms are shown in Fig. 2.

### 2.5 FRAP assay

The FRAP assay procedure is based on the method described by Benzie and Strain (1996), with some modification (Székelyhidi et al., 2024; Williams et al., 2022). 50 μL of supernatant, 3 mL of FRAP solution, and 100 μL of water were pipetted into a test tube. The finished solutions were placed in a dark place for 5 min and then the absorbance was measured with a Spectroquant Pharo 100 spectrophotometer (Merck, Darmstadt, Germany) at a wavelength of 593 nm against the blank. Ascorbic acid (40–500 mg/L) was used as a standard, the results were calculated according to Eq. 1 and were expressed as ascorbic acid equivalent (AAE)/ kg.

(1) $$c=\frac{V*A}{a*m}$$

c: the concentration.A: the absorbance of the component.V: dilution volume of sample solution.m: mass of the sample.a: slope of analytical measuring curve.

### 2.6 Folin-Ciocalteu assay

Determination of total polyphenol content based on the Folin-Ciocalteu method described by Singleton et al. (1999) with some modifications (Székelyhidi et al., 2024; Williams et al., 2022). To 50 μL of supernatant, 1.5 mL of high-purity water was pipetted and the reagents were added. First 2.5 mL of 10 % Folin-Ciocalteu reagent, then 2 mL of 7,5 % Na_2_CO_3_. The tubes containing the mixture were placed in a dark place for 90 min, and then the absorbance was measured at 750 nm versus the blank. Gallic acid was used as a standard (25–1000 mg/L), and the results were expressed as gallic acid equivalent (GAE)/ kg.

### 2.7 Melting rate

Determination of melting rate based on the method described by Muse and Hartel (2004) with some modifications (Williams et al., 2022). To assess the melting rate of sorbets, the frozen products (−18 °C) were taken out of the containers and 50 g placed on a wire rack with a mesh size of 1 cm^2^ above a glass funnel in a temperature-controlled room that was kept at 22.5 ± 0.5 °C. Up to 180 min, the weight of dripped sorbet was measured and recorded every 5 min. The melting rate was calculated and expressed as g/min after the dripped weight (g) was plotted against the time (min).

### 2.8 Consumer acceptance of sorbets

40 panelists (average consumers with average sensory sensitivity), including 25 women and 15 men, who are employees and students at the Albert Kázmér Faculty of Mosonmagyaróvár, at Széchenyi István University, evaluated from 1 to 5 the sorbet samples (5 meant they liked the samples very much, 1 meant they disliked it very much, 3 meant they neither liked nor disliked the sorbets). The average age of the respondents was 26. During the experiment, all regulations were followed, and consent was sought and obtained from all panelists, the appropriate protocols for protecting the rights and privacy of all participants were utilized during the execution of the research. Sensory tests did not require ethical approval. The panelists evaluated the samples using a questionnaire. The sensory properties of the sorbet samples: appearance, odor, taste, texture, and overall acceptability. The sorbets could be seen by the panelists in the freezing containers. The samples were provided for judging in a white plastic cup, and each sample was presented simultaneously to allow for proper comparison. The average scores for each parameter and the standard deviations were used to present the results.

### 2.9 Data analysis

The organic acid, sugar, total antioxidant, and polyphenol contents of strawberry sorbet samples were determined in Microsoft Office Excel from the values measured for the samples using the equation of the second-order least squares analytical curve fitted to the measurement solutions using the nonlinear least-squares method. All measurements were performed in triplicate (*n* = 3), and the results are expressed as the mean standard deviation (SD). Analyses of variance (ANOVA) followed by the Tukey post hoc test were used to compare the significant differences in the data. Differences were considered statistically significant when *p* < 0.05. The statistical analyses were carried out with Microsoft Excel 2016 software.

## 3 Results and discussion

### 3.1 Organic acid, sugar, total antioxidant (TAC), and polyphenol (TPC) content of raw materials

The organoleptic properties of fruits are significantly influenced by the ratio of organic acids and soluble sugars. According to Table 1, regarding the examined organic acids, lemon had the highest malic and citric acid concentration, followed by strawberries, which also contained smaller amounts of succinic acid. Only succinic acid, at 23164.22 ± 381.24 mg/kg, was present in the honey among the tested organic acids. The results of the sugar determination are also shown in Table 1. Based on the presented data, it can be seen that the fruits used for sorbet production contained only glucose and fructose. At the same time, small amounts of sucrose were also detected in the honey in addition to these components.

**Table 1:** Organic acid, sugar, total antioxidant (TAC), and total polyphenol (TPC) content of the raw materials (*n* = 3), different letters (a, b, c, d, and e) denote significant differences (*p* ≤ 0.05).

Parameters	Raw materials				
	Strawberry	Peppermint (dw)	Spearmint (dw)	Lemon	Honey
Organic acids (mg/kg)					
Malic acid	5101.67 ± 87.62^a^	n.d.	n.d.	23,724.58 ± 358.62^b^	n.d.
Succinic acid	689.76 ± 33.94^a^	n.d.	n.d.	n.d.	n.d.
Citric acid	6832.64 ± 116.66^a^	n.d.	n.d.	26,217.28 ± 1638.72^b^	n.d.
Sugars (mg/kg)					
Saccharose	n.d.	n.d.	n.d.	n.d.	3932.64 ± 601.99^a^
Glucose	22,590.09 ± 81.70^a^	n.d.	n.d.	34,669.04 ± 396.02^b^	252,235.84 ± 3072.41^c^
Fructose	25,078.59 ± 48.91^a^	n.d.	n.d.	30,521.64 ± 248.11^b^	398,727.97 ± 1167.20^c^
Antioxidant / phenolic components					
TAC (mg AAE/kg)	2037.08 ± 53.07^a^	52,708.30 ± 4533.45^b^	87,750.00 ± 9021.99^c^	291.88 ± 3.33^d^	115.83 ± 30.61^e^
TPC (mg GAE/kg)	1071.01 ± 48.35^a^	13,695.65 ± 1423.98^b^	22,608.70 ± 676.66^c^	607.67 ± 29.40^d^	37.65 ± 2.87 ^e^

n.d.: not detected.

The dominant organic acids in strawberry are citric and malic acid. The relative ratio of these two acids varies continuously during ripening, and at full maturity, it ranges between 1.2 and 3.1 (Milosavljević et al., 2023). Smaller ratios result in more evenly distributed citric and malic acid in the fruit. In the sorbet-making process, the strawberry sample had a ratio of 1.33.

According to several studies, citric acid is the dominant organic acid in citrus fruits and lemons (Liu et al., 2021; Xie et al., 2022). On the other hand, based on the organic acid composition of the lemon sample, the proportion of citric acid and malic acid was nearly equal. This finding is supported by Asencio et al.'s (2018) study, which examined the chemical composition of traditional Spanish citrus fruits and discovered that malic acid predominates in some lemon varieties in addition to citric acid.

The glucose and fructose content was above 60 g/100 g, meeting European Legislation requirements. The investigated acacia honey sample contained less than 5 g/kg of sucrose, which is the maximum permitted level under European legislation (European Union Directive (EU), 2002).

The samples of the dried mint had the highest concentration of phenolic (13,695.65 ± 1423.98–22,608.70 ± 676.66 mg GAE/kg), and antioxidant (52,708.30 ± 4533.45–87,750.00 ± 9021.99 mg AAE/kg) compounds (spearmint, peppermint). Many illnesses, such as irritable bowel syndrome, diarrhea, breast tenderness, dyspepsia, headaches, abdominal distention, abdominal pain, and bad breath, have been treated medicinally with mentha species (Anwar et al., 2019). The Mentha genus of medicinal plants contains a variety of compounds, including carotenoids, ascorbic acid, and phenolic compounds, which can prevent or delay the oxidation of different molecules (Tafrihi et al., 2021., Bouzid et al., 2023). Studies on spearmint's antioxidant qualities have been conducted in large numbers, but the results have varied greatly. The antioxidant content of dried spearmint leaves was measured by Henao-Rojas et al. (2022) with 19.63 mg AAE/g value, compared to 33.96 mg AAE/g by Direito et al. (2019). The literature also describes significantly different TAC values of peppermint. Hazarika and Gosztola (2022) determined an antioxidant content of 210.2 mg AAE/g, whereas Oh et al. (2013) only measured 40.70 mg AAE/g.

Data from the literature on the polyphenol content of spearmint also revealed notable variations. Henao-Rojas et al. (2022) reported values of 11.83 and 17.81 mg GAE/g, whereas Kaloteraki et al. (2021) measured 39.61 mg GAE/g. Oh et al. (2013) found that the polyphenol content of peppermint was 33.68 mg GAE/g. Jeong et al. (2018) found that peppermints from different countries had polyphenol contents ranging from 55.23 to 98.27 mg GAE/g. The production of secondary metabolic products of medicinal plants is highly dependent on environmental factors (temperature, hours of sunshine, UV radiation level, soil conditions, rainfall), which explains the notable discrepancies in the literature data (Pant et al., 2021).

Following the mints, the strawberry that served as the sorbet's base had a significant TAC (2037.08 ± 53.07 mg AAE/kg) and TPC (1071.01 ± 48.35 mg GAE/kg) level.

The lemon added to preserve the red color of the strawberries and the acacia honey added to achieve an appropriately sweet taste had significantly less TAC and TPC content than the other tested ingredients. Rekha et al. (2012) examined the juice of ripe and unripe citrus fruits and determined that the juice of unripe fruits contains higher amounts of antioxidant and phenolic compounds. In the case of lemon juice, a total polyphenol content of 600 (ripe) - 760 (unripe) mg GAE/L was determined, which is the same as the presented measurement result (607.67 ± 29.40 mg GAE/L). In contrast, Oboh et al. (2015) found significantly lower amounts of TPC (64.5 ± 1.4 mg GAE/L) and TAC (11.85 mg AAE/L) content during the analysis of lemon juices. Honey's health-promoting properties are due to the presence of phenolic compounds such as flavonoids, phenolic acids and their esters, and organic acids. These compounds act as antioxidants, actively protecting biological compounds from oxidation (Kędzierska-Matysek et al., 2021). Di Marco et al. (2018) investigated the antioxidant compounds of Italian honey samples and determined a TPC content of 107.2 ± 35.7 mg GAE/kg and a TAC content of 27.8 ± 3.1 μmol AAE/g in the case of acacia honey. In contrast, Gül and Pehlivan (2018) examined Turkish monofloral honey samples and detected 51.91 mg GAE/100 g TPC and 12.72 mg/mL TAC content in acacia honey. Honey's phenol composition is primarily determined by its botanical origin, and the amount of phenolic compounds varies depending on the season, climatic conditions, and processing factors. Honey's complex matrix, low concentrations of these compounds, and differences in the analysis and presentation make it difficult to compare results (Pauliuc et al., 2020).

### 3.2 Organic acid, sugar, total antioxidant (TAC), and polyphenol (TPC) content of sorbet samples

Strawberries are widely consumed both fresh and processed, but the quality of the fruit is significantly affected by environmental effects, degree of maturity and storage conditions (Tulipani et al., 2011). Hipólito et al. (2016) found in their research that the origin of the fruit greatly influences its quality and medium-sized, hard-textured, and bright red fruits are needed to make high-quality strawberry sorbet.

Table 2 shows the test results of the chemical composition of the sorbets. The malic acid content of the sorbets with 1, and 2 % peppermint and 0.5 %, 1 %, and 2 % spearmint increased significantly compared to the control sorbet. No significant difference was detected in the case of 0.5 % peppermint dosage. The succinic acid content of experimental samples was not affected by the addition of peppermint, however, with the addition of spearmint, a significant decrease was measured in all cases compared to the control sample. The citric acid content of the sorbets was not affected by the addition of mint species.

**Table 2:** Organic acid, sugar, total antioxidant (TAC), and total polyphenol (TPC) content of the sorbet samples (n = 3), different letters (a, b, c, d, and e) denote significant differences (p ≤ 0.05).

Parameters	Sorbet samples						
	Control	Peppermint 0.5 %	Peppermint 1 %	Peppermint 2 %	Spearmint 0.5 %	Spearmint 1 %	Spearmint 2 %
Organic acids (mg/kg)							
Malic acid	5730.85 ± 126.62^a^	5789.26 ± 212.79^a,b^	6200.36 ± 209.62^b,d^	6736.88 ± 180.30^c^	6218.17 ± 194.30^d^	6709 ± 260.93^c^	6952.03 ± 293.67^c^
Succinic acid	4029.49 ± 258.68^a^	3783.13 ± 182.64^a^	3972 ± 68.34 ± 68.34^a^	4055.80 ± 487.86^a^	2408.34 ± 47.21^b^	2369.26 ± 60.12^b^	2378.09 ± 48.72^b^
Citric acid	4238.15 ± 225.97^a^	4125.02 ± 252.41^a^	4434.73 ± 100.80^a^	4443.18 ± 164.13^a^	4246.01 ± 178.41^a^	4517.21 ± 195.78^a^	4342.21 ± 192.01^a^
Sugars (mg/kg)							
Saccharose	7932.17 ± 149.58 ^a^	10,041.83 ± 284.52^b^	8786.26 ± 284.52^c^	6525.43 ± 142.61^d^	8551.52 ± 336.33^c^	10,083.75 ± 217.65^b^	10,769.48 ± 149.56^b^
Glucose	41,663.47 ± 1581.91^a^	55,190.18 ± 1592.46^b^	49,330.09 ± 1644.89^c^	41,224.31 ± 758.74^a^	49,726.24 ± 1774.13^c^	58,408.70 ± 1472.49^d^	61,891.27 ± 898.22^e^
Fructose	41,663.47 ± 1581.92^a^	60,220.82 ± 1729.75^b^	53,800.07 ± 1760.63^c^	45,186.00 ± 895.59^a^	53,311.24 ± 771.64^c^	63,356.24 ± 1423.59^b^	67,614.98 ± 990.25^d^
Antioxidant / phenolic components							
TAC (mg AAE/kg)	946.00 ± 77.23^a,b^	906.64 ± 42.29^a^	940.64 ± 34.10^a,b^	1000.00 ± 35.90^b,d^	1115.67 ± 42.49^c^	1092.00 ± 18.11^c^	1137.67 ± 115.42^c,d^
TPC (mg GAE/kg)	625.65 ± 34.70^a^	510.72 ± 19.94^b^	696.67 ± 30.55^c,d^	622.61 ± 45.47^a,c^	725.80 ± 59.41^c,d^	666.23 ± 13.62^c^	743.77 ± 55.28^d^

In every instance, the addition of 0.5 % and 1 % peppermint and spearmint increased the sorbets' sucrose, glucose, and fructose content. Compared to the control sample, adding 2 % peppermint significantly decreased the sucrose content while not affecting the glucose or fructose content. There are no comprehensive chemical test results for sorbets that can be used to compare the results, but according to Rachmayati et al. (2017), a product's antioxidant content was impacted by the addition of sugar in addition to the fruit's ripeness. Vitamin C is one of the antioxidant sources of sorbet, and it's oxidized by sugar, so the higher sugar content resulting lower antioxidant content. Although mint is an excellent source of antioxidants, it increased the sugar content in the sorbets and the oxidation of vitamin C could be the reason that the amount of antioxidant compounds in the products increased to a lesser extent than expected.

Based on the data in Table 2, the TAC content shows that adding 2 % peppermint and 0.5 % 1 % 2 % spearmint significantly increased the antioxidant content of the experimental samples compared to the control sample. The sorbets' TAC content was unaffected by adding peppermint at 0.5 % and 1 %. Petkova et al. (2022) wanted to increase the TAC and TPC content of peach sorbets by adding Zizyphus jujuba and *Stevia rebaudiana*, but the used plants acted as prooxidants in the matrix.

Regarding the TPC contents, only the 1 % peppermint addition could significantly increase the total polyphenol content of the sorbets. In the case of 0.5 % peppermint dosage, the TPC value decreased, while in the case of 2 % dosage, it did not change compared to the control sample. Conversely, when spearmint was added to the products, the sorbets' TPC content increased significantly in all used doses. Compared to the results of fresh strawberries, the antioxidant and polyphenol content of the control sorbet decreased significantly. Since no water was added to the products during the preparation of the sorbets, the significant reduction can be attributed to the processing. This is supported by Hipólito et al. (2016) study, in which it was established that fruits' TPC and TAC content decrease during processing.

### 3.3 Results of sorbet melting parameters

**Fig. 3:**
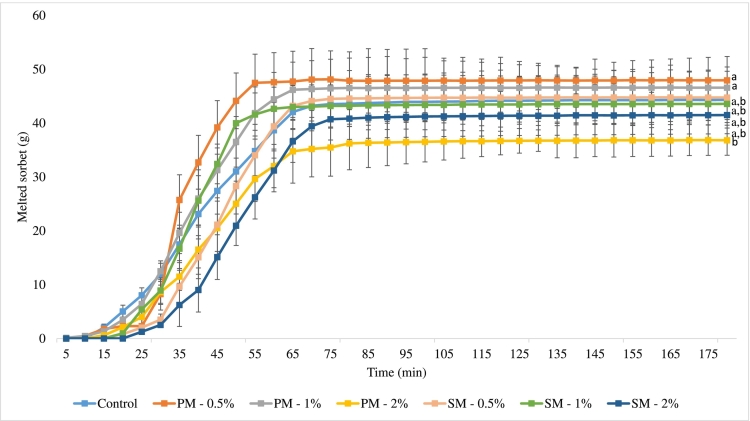
The melting rate of the strawberry sorbet samples at 22 °C (n = 3), different letters (a, and b) denote significant differences (p ≤ 0.05) in the average melting rate of sorbets.

When assessing the quality of frozen desserts, melting time is a crucial factor. Several factors, including overrun, total dissolved solids, air content, viscosity, ice crystal size, acidity, and stabilizers used in the manufacturing of ice cream products, can affect melting rate which impact product quality and consumer perception (Freire et al., 2020; Putradamni & Pramitasari, 2024; Ranaweera et al., 2022). Fig. 3 shows the test results of the melting rate of the sorbets. The sorbets began to drip intensively from the 15th minute of the melting test. Overall, it can be said that compared to the control sample (average melting rate: 0.19 ± 0.02 g/min), the experimental samples containing 2 % mint melted more slowly. Due to the addition of mint, the dry matter content of the sorbets increased and thus the melting speed decreased. Several studies mention the effect of the dry matter content of sorbets on the melting parameters (de Medeiros et al., 2019; Kilicli et al., 2025). The sorbet containing 2 % peppermint melted at an average rate of 0.16 ± 0.02 g/min, while the 2 % spearmint sorbet melted at 0.17 ± 0.02 g/min. The average melting rate of sorbets containing 0.5 and 1 % spearmint was 0.19 ± 0.02 g/min, while the average melting value of sorbets containing 0.5 % peppermint was 0.22 ± 0.02 g/min, and for 1 % peppermint sorbet it was 0.21 ± 0.02 g/min. A significant difference in the average melting rate was found only between the values of the 0.5 and 1 % peppermint sorbets, and the 2 % spearmint sorbet. The melting parameters were also influenced by the fact that, compared to the control sorbets, the addition of mint increased the sugar content of the products. According to research by Seftiono et al. (2020), the higher the sucrose content, the longer the melting time because sucrose can bind water in a product. Patil et al. (2018) research shows that the viscosity and overrun of ice creams decrease due to the addition of mint, in the case of sorbets such a significant effect cannot be inferred based on the melting rates. When examining industrial and crafted sorbets, Agnieszka and Wilczyńska (2023) found that the melting speed of crafted sorbets was much higher than that of industrial sorbets, which was probably the result of the high fruit content of all mixtures and the natural stabilizer effect of pectin. According to Bohra (2023), the melting rate of the flash-frozen crafted ice cream samples ranged between 0.1 and 0.8 g/min, compared to 0.2 g/min for commercial premium ice cream. Based on these, the melting properties of the sorbets examined in this study were similar to the industrial sorbets.

### 3.4 Results of consumer acceptance of sorbets

**Fig. 4:**
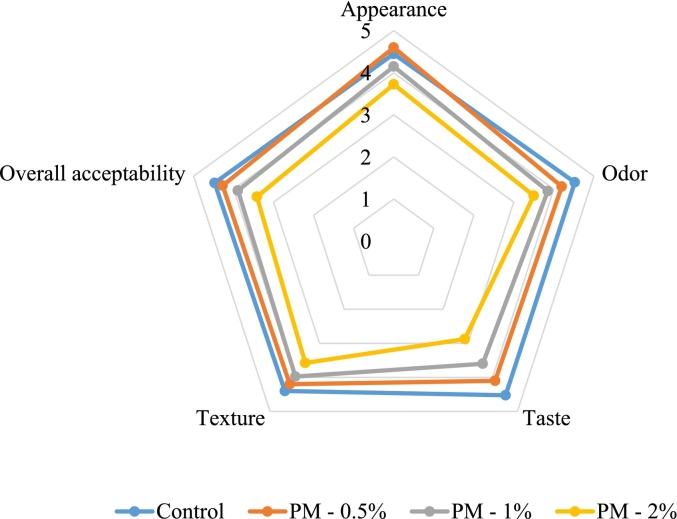
Consumer acceptance evaluation of strawberry sorbets with peppermint supplementation; PM – 0.5 % - enriched with 0.5 % peppermint powder, PM – 1 % - enriched with 1 % peppermint powder, PM – 2 % - enriched with 2 % peppermint powder (*n* = 40).

**Fig 5:**
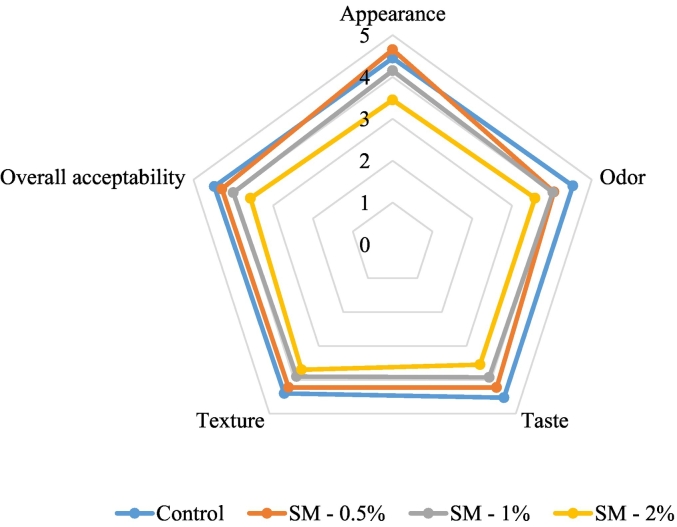
Consumer acceptance evaluation of strawberry sorbets with spearmint supplementation; SM – 0.5 % - enriched with 0.5 % spearmint powder, SM – 1 % - enriched with 1 % spearmint powder, SM – 2 % - enriched with 2 % spearmint powder (n = 40).

Figs. 4 and 5 show the results of consumer acceptance of peppermint and spearmint sorbets. Overall, the control (4.50), and the sorbets with 0.5 % peppermint (4.28) and spearmint (4.29) addition received the highest acceptance scores. In terms of appearance (control: 4.45, peppermint 0.5 %: 4.60, spearmint 0.5 %: 4.65) and texture (control: 4.40, peppermint 0.5 %: 4.20, spearmint 0.5 %: 4.23), the panelists also rated these samples as the best. The panelists concluded that the sorbets' sensory qualities were diminished by the addition of 1 or 2 % mint in each case because the flavor was similar to strongly menthol toothpaste. In terms of appearance, the sorbet supplemented with 0.5 % peppermint outperformed the control, but it lost points in every other category. Though there isn't much of a market for menthol confections in Hungary - menthol is usually used in pharmaceutical, and oral hygiene products - a number of panelists thought the 0.5 % peppermint sorbet tasted, smelled, and texture better than the control. In Great Britain, where menthol chocolates, ice creams, candies, etc. are widely available, such a product would be more well-liked.

## 4 Conclusion

The need to create novel functional food forms arises from the ongoing shift in consumer preferences. Enriching sorbets with herbs is an excellent alternative to increase the functionality of these popular cooling desserts. Based on the results, the addition of spearmint and peppermint significantly increases the TAC, and TPC content of the sorbets. Antioxidant, anticarcinogenic, immunomodulatory, anti-inflammatory, antiallergenic, antiviral, and antibacterial are just a few of the numerous well-known beneficial physiological effects of these active ingredients that support human health preservation. Among the water-soluble sugars, fructose and glucose are found in greater amounts in sorbets; consuming these two sugars together can improve physical performance and reduce fatigue. Both varieties of used mint raised malic acid content among the organic acids found in the products, while spearmint reduced the succinic acid content. Malic acid has a beneficial effect on the functioning of the intestinal system and liver, reducing the risk of kidney stones and uric acid formation. The addition of mint did not affect the melting properties of the sorbets, which was similar to premium frozen desserts. Based on consumer feedback, 0.5 % of spearmint and peppermint resulted in sorbets with accepted organoleptic properties. During the development of herbal functional products, in addition to the positive change in content values, care must be taken to ensure that the products also meet consumer needs in terms of their organoleptic properties.

## CRediT authorship contribution statement

**Rita Székelyhidi:** Writing – review & editing, Writing – original draft, Methodology, Conceptualization. **Erika Lakatos:** Resources. **Zsófia Tóth:** Investigation, Data curation. **Beatrix Sik:** Methodology, Conceptualization.

## Declaration of competing interest

The authors declare that they have no known competing financial interests or personal relationships that could have appeared to influence the work reported in this paper.

## Data Availability

No data was used for the research described in the article.

## Appendix A Supplementary data

Supplementary material Image 1
