# Supplementary material for: The effect of mint addition on the physicochemical and organoleptic properties of strawberry sorbets
Source: Food Chem X. 2025 Feb 12;26:102271. doi: 10.1016/j.fochx.2025.102271 (PMC11872461; doi:10.1016/j.fochx.2025.102271)
Supplement: Supplementary file 1 — Supplementary material [file mmc1.pdf]

Ethical Statement for Food Chemistry: X

Hereby, I /insert author name/ consciously assure that for the manuscript /insert title/ the following is fulfilled:

1) This material is the authors' own original work, which has not been previously published elsewhere.

2) The paper is not currently being considered for publication elsewhere.

3) The paper reflects the authors' own research and analysis in a truthful and complete manner.

4) The paper properly credits the meaningful contributions of co-authors and co-researchers.

5) The results are appropriately placed in the context of prior and existing research.

6) All sources used are properly disclosed (correct citation). Literally copying of text must be indicated as such by using quotation marks and giving proper reference.

7) All authors have been personally and actively involved in substantial work leading to the paper, and will take public responsibility for its content.

The violation of the Ethical Statement rules may result in severe consequences.

To verify originality, your article may be checked by the originality detection software iThenticate. See also http://www.elsevier.com/editors/plagdetect.

I agree with the above statements and declare that this submission follows the policies of Solid State Ionics as outlined in the Guide for Authors and in the Ethical Statement.

Date: 07. 10. 2024

Corresponding author's signature:
